# Investigation of fMRI activation in the internal capsule

**DOI:** 10.1186/1471-2202-12-56

**Published:** 2011-06-14

**Authors:** Jodie R Gawryluk, Erin L Mazerolle, Kimberley D Brewer, Steven D Beyea, Ryan CN D'Arcy

**Affiliations:** 1 Institute for Biodiagnostics (Atlantic), National Research Council, Halifax, Nova Scotia, Canada; 2 Department of Psychology/Neuroscience, Dalhousie University, Halifax, Nova Scotia, Canada; 3 Department of Physics and Atmospheric Sciences, Dalhousie University, Halifax, Nova Scotia, Canada; 4 Department of Radiology, Dalhousie University, Halifax, Nova Scotia, Canada

## Abstract

**Background:**

Functional magnetic resonance imaging (fMRI) in white matter has long been considered controversial. Recently, this viewpoint has been challenged by an emerging body of evidence demonstrating white matter activation in the corpus callosum. The current study aimed to determine whether white matter activation could be detected outside of the corpus callosum, in the internal capsule. Data were acquired from a 4 T MRI using a specialized asymmetric spin echo spiral sequence. A motor task was selected to elicit activation in the posterior limb of the internal capsule.

**Results:**

White matter fMRI activation was examined at the individual and group levels. Analyses revealed that activation was present in the posterior limb of the internal capsule in 80% of participants. These results provide further support for white matter fMRI activation.

**Conclusions:**

The ability to visualize functionally active tracts has strong implications for the basic scientific study of connectivity and the clinical assessment of white matter disease.

## Background

White matter represents approximately half of the tissue in the brain [1]. The idea of white matter activation in fMRI represents an important advance for both basic and clinical studies. Enabling the measurement of functional connectivity more directly than current fMRI approaches, white matter fMRI could provide valuable insight into the dynamics of distributed neural systems and white matter diseases.

However, white matter fMRI is a controversial idea and, up until recently, has been largely disregarded in the literature. The majority of fMRI studies have restricted their focus to gray matter for two reasons; 1) the BOLD signal relies on cerebral blood volume and flow which are 3-6 times lower in white matter [2-6], and 2) the primary source of fMRI signal is thought to arise from post-synaptic potentials as opposed to action potentials [7]. Despite these arguments, white matter tissue has metabolic demands that must be met. This fact has given rise to the idea that white matter activation may be detectable using fMRI. Indeed, an increasing number of fMRI studies have shown white matter activation [8-17].

Most of the white matter fMRI reports have employed tasks that exploit the lateralized nature of the visual and motor systems (opposite response hand to visual hemifield presentation creates a so-called 'crossed' condition) to study interhemispheric transfer and related information processing phenomena. Recently, our group reported prospective white matter fMRI studies at 4T using tasks designed to elicit interhemispheric transfer across the corpus callosum. Mazerolle et al. used a visual Sperry task (word/face) to detect activation in the isthmus of the corpus callosum [13,18]. These results were observed at the group level and in 20% of the individual subjects (N = 24, p < 0.005, uncorrected). Gawryluk et al. used a Poffenberger task (visual/motor) to elicit activation in the anterior corpus callosum [15,19]. These results were present at the group level and, notably, in 100% of the individual subjects (N = 10, p < 0.05 corrected).

A key factor that accounted for the sensitivity difference between the two studies related to the method of acquisition. Gawryluk et al. [15] sought to enhance the detection of white matter fMRI using a novel imaging sequence called asymmetric spin echo (ASE) spiral [20]. ASE spiral acquires three images (per slice per volume) with increasing T_2_-weighting but equal BOLD-contrast. Sensitivity to white matter fMRI activation increased with increasing T_2_-weighting, with the third ASE spiral image (with the highest T_2_-weighting) the demonstrating a significant increase in percent signal change relative to the first ASE spiral image (with the lowest T_2_-weighting). Moreover, the extent of active voxels in white matter increased as a function of T_2_-weighting. The results provided valuable insight into optimizing fMRI acquisition for the detection of white matter activation. Indeed, in a subsequent within-subjects study that administered both the Sperry and Poffenberger tasks using the ASE spiral method of acquisition, corpus callosum activation was observed in 100% and 94% of participants, respectively [17]. These results provide further evidence of the sensitivity of ASE spiral to the detection of white matter fMRI.

There is additional evidence favoring the detection of fMRI signal in white matter. First, white matter activation appears to improve when motion is included as a regressor in the model [13,17]; this would not be expected if the activation resulted from motion artifact. Second, recent work has shown that white matter activation varies according to task type, indicating that it can be functionally manipulated [17]. Third, as Mazerolle et al. demonstrated, diffusion tensor imaging (DTI) based tractography data can be used to confirm structural connections between active regions in gray and white matter [16].

Taken together, the research to-date has reported white matter activation in the corpus callosum. However, in these previous studies, we cautioned that it is important to verify white matter activity in other structures [13,15,17]. This is particularly important if the intent is to develop future applications in both basic science (e.g. the study of functional connectivity) as well as clinical practice (e.g. the assessment of white matter diseases). To extend the current findings, we examined the possibility of detecting fMRI activation in another white matter structure, namely the internal capsule.

To date, the only evidence for white matter fMRI in a fibre tract other than the corpus callosum comes from an abstract by Maldjian et al. [21]. The protocol involved motor tasks with data collected at 1.5 and 4 T. White matter activation was observed in the posterior limb of the internal capsule (PLIC) at 4 T only.

Given that more evidence is needed to characterize controversial white matter activation in pathways outside the corpus callosum, the current study sought to answer the following question: Can white matter fMRI activation be detected in the PLIC using a basic motor paradigm at 4 T? Accordingly, it was hypothesized that it is possible to detect white matter fMRI activation in the PLIC at both the individual and group level.

## Results

**Figure 1:**
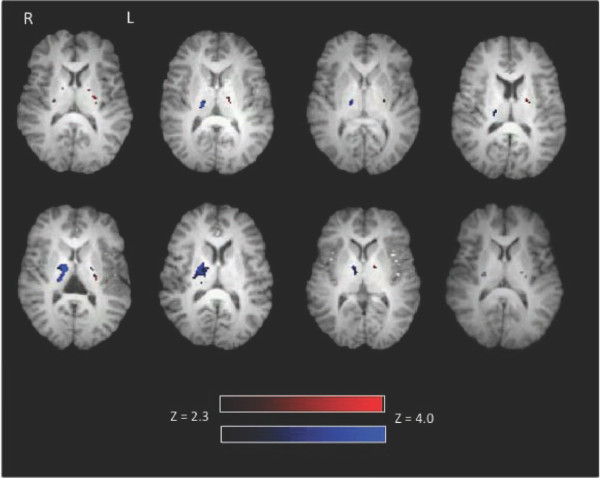
**Individual level activation during right and left finger tapping**. Eight out of ten participants showed activation in the PLIC. Left finger tapping related activation (displayed in blue) is present in the right posterior limb of the internal capsule. Right finger tapping related activation (displayed in red) is present in the left posterior limb of the internal capsule. Of the eight participants with PLIC activation, 100% had activation in the right PLIC and 87.5% showed activation in the left PLIC. Activation intensity is displayed in terms of Z-scores with a Z threshold of 2.3.

At the individual level, PLIC activation was present in 80% of participants (8/10). The left finger tapping condition elicited activation in the right PLIC in 100% of these participants (8/8). The right finger tapping condition elicited activation in the left PLIC in 87.5% of these participants (7/8). Figure 1 shows activation in the PLIC in each subject for each condition. Table 1 reports the maximum Z score and peak coordinates in the PLIC for each participant.

**Table 1:** The maximum Z score and peak co-ordinates (MNI space) in the posterior limb of the internal capsule at the individual and group levels during right finger tapping (left hemisphere) and left finger tapping (right hemisphere).

Subject	Hemisphere	Maximum Z score	Co-ordinates of maximum (MNI)
1	Left	3.63	-24 -6 14
	Right	3.62	26 -14 8
2	Left	3.64	-14 -12 8
	Right	3.66	14 -12 8
3	Left	2.85	-16 -2 6
	Right	4.23	18 -20 2
4	Left	3.83	-28 -16 16
	Right	3.80	20 -22 16
5	Left	4.10	-24 -26 14
	Right	5.00	14 -10 6
6	Right	4.05	20 -2 10
7	Left	4.28	-14 -2 0
	Right	4.72	18 -18 4
8	Left	3.31	-22 -12 -2
	Right	3.84	26 -14 6
GROUP	Right	3.15	20 -22 14

**Figure 2:**
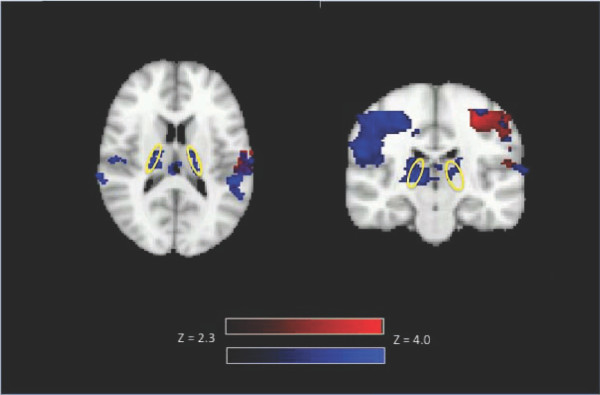
**Group activation (N = 10) during right finger tapping (displayed in red) and left finger tapping (displayed in blue)**. White matter activation is present in the right and left posterior limb of the internal capsule during left hand finger tapping (blue). Activation is over-laid on the standard brain in axial (slice 45) and coronal (slice 54) view. Activation is displayed with a Z threshold of 2.3.

The task also showed group level activation in the PLIC (Figure 2; Table 1). The left finger tapping condition produced activation in the right PLIC and to a lesser extent the left PLIC. Conversely, the right finger tapping condition did not reveal PLIC activation.

**Figure 3:**
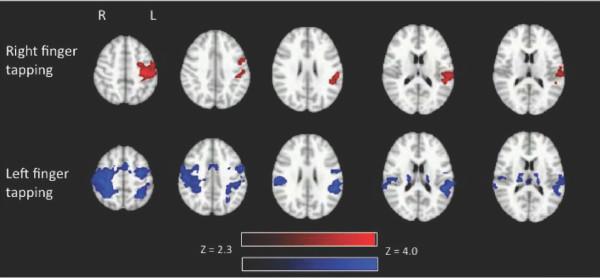
**Group activation (N = 10) in white and gray matter during right finger tapping (above) and left finger tapping (below)**. Activation related to right finger tapping is displayed in red and activation related to left finger tapping is displayed in blue. Activation is present in the left primary motor cortex during right finger tapping. Activation is present in the right and left posterior limb of the internal capsule and right primary motor cortex during left finger tapping. Activation is displayed with a Z threshold of 2.3.

Additional group level white matter activation was present in the superior longitudinal fasciculus (bilaterally) for both conditions. Gray matter task related activation was present in frontal (precentral gyrus, middle frontal gyrus), parietal (postcentral gyrus, superior parietal lobule, inferior parietal lobule, precuneus), temporal (superior temporal gyrus), and subcortical (basil ganglia, thalamus) regions and in the insula in the left hemisphere for the right finger tapping condition and bilaterally for the left finger tapping condition. The left tapping condition also elicited limbic (cingulate) activation. Figure 3 shows the above group results.

## Discussion

The current study examined whether white matter fMRI activation could be reliably detected in the internal capsule using a basic motor task and 4 T imaging. As predicted, white matter fMRI activation was detected in the PLIC at both the individual and group level.

These findings correspond with fMRI results obtained by Maldjian et al. [21]. As mentioned, Maldjian et al. collected data at 1.5 and 4 T and observed activation in the PLIC at 4 T only, emphasizing the importance of high field strength to the detection of white matter fMRI [21].

The current findings are also consistent with known neuroanatomy. The PLIC contains corticospinal fibers and is thought to directly connect to the primary motor cortex [22]. Such connections have previously been demonstrated in healthy subjects using a combination of fMRI activation in the primary motor cortex and DTI tractography [23]. Given this, the finding of white matter fMRI activation in the PLIC is thought to be functionally consistent with the motor task employed. The most pronounced activation in the internal capsule was present in the hemisphere contralateral to the engaged hand (Figure 2). This pattern of activation (bilateral: contralateral > ipsilateral) matches that typically seen in the primary motor cortices for a finger tapping task.

One potential concern with imaging a structure as small as the internal capsule is partial volume effects resulting from limitations in resolution. Although the current study used higher resolution than previous work (thinner slices), resolution remains a limitation to be addressed in future research. To address concerns about partial voluming, we examined the individual level data for consistent localization to the PLIC using a double rater approach. Using this method, white matter activation in the PLIC was present in 80% of participants (Figure 1). This proportion of subjects with white matter activation is highly consistent with previous results [15,17]. This is likely due to the use of ASE spiral imaging in combination with high field MRI.

Interestingly, of participants with activation in the PLIC, 87.5% showed activation in the left hemisphere during right finger tapping and 100% showed activation in the right hemisphere during left finger tapping. Additionally, at the group level, PLiC activation was only observed during left finger tapping. Given that all participants were right handed, this difference may reflect task demand related to hand dominance.

A common question that has arisen is: why is activity not visible along the entire white matter tract? There are at least three key considerations related to this issue. First, it is important to emphasize that the objective of this work is to demonstrate functional MRI sensitivity to activation in white matter, which was previously thought to be undetectable. Given this, the initial evidence is expected to occur in the regions that show the strongest activation. Once sensitivity to the phenomenon is better understood, better visualization is expected. Second, related to point one, the most active regions along a tract can vary. In the current study, individuals showed variability in PLIC activation (Figure 1; Table 1). Evidence from studies on the vasculature supplying the PLIC corroborates these findings. Specifically Donzetli et. al. documented variability in vasculature feeding the region of the PLIC [24]. Third, if WM activity could be explained solely on the basis of false positives, when averaged together as a group, one would not expect a group representation of the white matter structure. Our data show clear a group effect, which in fact is an improved representation of the underlying anatomy (Figure 2). In order to further address the question, we are currently studying white matter activation in an animal model using a combination of cerebral blood flow/volume measurements and electrophysiological techniques.

Despite increasing evidence, the detection of fMRI in white matter remains controversial. However, studies using fluorodeoxyglucose autoradiography in rats have detected activity-dependent metabolic changes in white matter [25]. There is also evidence that spiking activity is correlated with fMRI activation in addition to local field potentials (albeit to a lesser extent) [26-28]. One contributing neurophysiologic source of fMRI signal changes in white matter is increased activity of ATP-dependent Na+/K+ pumps, required to restore ionic gradients that are disrupted by axonal conduction [29-31]. Further investigation of the physiological basis and possible imaging mechanism underlying this phenomenon are ongoing.

## Conclusions

Despite the fact that white matter comprises half of the brain, few studies have attempted to measure fMRI activity in this tissue. Recently, we reported fMRI activation in the corpus callosum [13,15-17]. The current study provides evidence of white matter fMRI activation in the internal capsule. These results represent an important avenue to advance in studies of functional connectivity as well as the clinical assessment of white matter disease/disorder.

## Methods

### Participants

Ten healthy, right handed subjects (five females) participated in the study. The mean age of the participants was 26.4 (SD = 5.2). The study was approved by the local ethics boards and each participant gave their informed consent prior to their participation.

### Experimental Design

The task was optimized based on the Maldjian et al. study [21]. Each participant performed a finger tapping task while holding a foam ball in each hand. All task instructions were presented visually via back-projection to a screen mounted inside the bore (and viewed through a mirror mounted on the head coil) using E-prime (Psychology Software Tools, Inc). The instructions indicated on and off blocks as well as which hand to tap with (order of left and right was randomized). The task consisted of 8 blocks (20s on, 20s off). Participants focused on a central fixation point during the rest phases. Each subject performed a short practice to ensure task compliance.

### Imaging Protocol

Data were acquired from a 4 T Varian INOVA whole body MRI system. Gradients were provided by a body coil (Tesla Engineering Ltd.) operating at a maximum of 35.5 mT/m at 120 T/m/s, and driven by 950 V amplifiers (PCI). The RF coil used was a TEM head coil (Bioengineering Inc.). All subjects underwent the same imaging protocol consisting of fMRI acquisition and a high-resolution T1 weighted scan. All images were obtained within one session that was approximately 60 minutes in duration.

### FMRI Acquisition

FMRI was conducted using the ASE spiral sequence [20]. The ASE spiral sequence collects three images (each with differing contrast) per slice per volume. Due to the parameters needed to collect three images per slice per volume, the number of slices was limited to 17 (4 mm axial slices, with no gap, interleaved). Slices were prescribed to cover the region extending from the internal capsule to the primary motor cortex using a 64 × 64 matrix (220 × 220 mm), with 1 shot and a volume repetition time of 2 s (170 volumes). The sequence had an asymmetric echo time of 27 ms, and a spin-echo centre of 68 ms.

### Structural Image Acquisition

Following the fMRI scans, a 3D MP FLASH T_1_-weighted whole brain anatomical scan (72 2 mm axial slices) was collected for registration purposes. The parameters were as follows: a repetition time of 10 ms, an inversion time of 500 ms, and an acquisition echo time of 5 ms.

### FMRI Data Analyses

Prior to data analyses, the three ASE images were combined using an inverted signal weighted averaging algorithm. This approach was taken based on the results of our previous study, which indicated that ASE spiral is more sensitive to white matter fMRI activation due to the T_2 _weighting of the third image [15].

Statistical analyses were performed using a model-based approach (General Linear Model) in FMRIB Software Library (FSL) using fMRI expert analysis tool (FEAT) version 5.3 (FMRIB's Software Library). Pre-statistics processing steps included motion correction using MCFLIRT [32], non-brain removal using BET [33], spatial smoothing using a Gaussian kernel of FWHM 5 mm, mean-based intensity normalization of all volumes by the same factor, and highpass temporal filtering (100 s cutoff). Time-series statistical analyses were carried out using FILM with local autocorrelation correction, motion included s a regressor, and a temporal derivative included in the model [34]. Activation was modeled as two boxcar functions representing the blocks of right and left finger tapping separately, convolved with a gamma function. Contrasts were calculated to statistically compare each finger tapping condition to rest. Z statistic images were developed using a threshold for clusters determined by Z > 2.3 and a (corrected) cluster significance threshold of P = 0.05 [35].

Registration was of particular importance in this study, given our small region of interest in the posterior limb of the internal capsule. To ensure the best possible registration, a variety of approaches were compared (e.g. functional images were registered to high resolution anatomical images as well as to standard images with different contrasts). The optimal registration method was subsequently employed; images were registered to the SPM EPI template (12 DOF) before being registered to the standard MNI152 image (12 DOF) using FLIRT [32,36]. The two-step registration approach is standard in FSL; in this case, using an image of comparable contrast to the functional data (the SPM EPI template) for the initial registration, improved the registration to standard space. Additionally, the accuracy of registration was manually confirmed for each subject (by JG and EM (neuroscientists)). Data were analyzed at the individual and group levels. The JHU ICBM-DTI White Matter Labels of the left and right PLIC were then combined with activation maps for each participant. PLIC activation was also manually verified for each subject (by JG and EM).

## Authors' contributions

JG was involved in the conceptualization and design of the study, acquisition of data, analysis and interpretation of data and drafting the manuscript. EM was involved in the acquisition of data, analysis and interpretation of data and revising the manuscript. SB and RD were involved in the acquisition of data and critically revised the manuscript for intellectual content. All authors read and approved the final manuscript.
